# Possibility of cancer-stem-cell-targeted radioimmunotherapy for acute myelogenous leukemia using ^211^At-CXCR4 monoclonal antibody

**DOI:** 10.1038/s41598-020-63557-9

**Published:** 2020-04-22

**Authors:** Noboru Oriuchi, Miho Aoki, Naoyuki Ukon, Kohshin Washiyama, Chengbo Tan, Saki Shimoyama, Ken-ichi Nishijima, Kazuhiro Takahashi, Hiroshi Ito, Takayuki Ikezoe, Songji Zhao

**Affiliations:** 1 Advanced Clinical Research Center, Fukushima Global Medical Science Center, Fukushima Medical University, Fukushima, 960-1295 Japan; 2 Department of Nuclear Medicine, Fukushima Medical University, Fukushima, 960-1295 Japan; 3 Department of Radiology, Fukushima Medical University, Fukushima, 960-1295 Japan; 4 Department of Hematology, Fukushima Medical University, Fukushima, 960-1295 Japan

**Keywords:** Oncology, Stem cells

## Abstract

To explore stem-cell-targeted radioimmunotherapy with α-particles in acute myelogenous leukemia (AML), pharmacokinetics and dosimetry of the ^211^At-labeled anti-C-X-C chemokine receptor type 4 monoclonal antibody (^211^At-CXCR4 mAb) were conducted using tumor xenografted mice. The biological half-life of ^211^At-CXCR4 mAb in blood was 15.0 h. The highest tumor uptake of 5.05%ID/g with the highest tumor-to-muscle ratio of 8.51 ± 6.14 was obtained at 6 h. Radiation dosimetry estimated with a human phantom showed absorbed doses of 0.512 mGy/MBq in the bone marrow, 0.287 mGy/MBq in the kidney, and <1 mGy/MBq in other major organs except bone. Sphere model analysis revealed 22.8 mGy/MBq in a tumor of 10 g; in this case, the tumor-to-bone marrow and tumor-to-kidney ratios were 44.5 and 79.4, respectively. The stem-cell-targeted α-particle therapy using ^211^At-CXCR4 mAb for AML appears possible and requires further therapeutic studies.

## Introduction

Targeted α-particle therapy (TAT) has great potential for the treatment of cancer based on the specific delivery of a high radiation dose from α-particles emitting radionuclides to tumors while minimizing systemic toxic effects, and it may lead to additional treatment options for many types of advanced or refractory cancer^1^. The high level of radiobiological effectiveness of α-particles, in comparison with β-particles emissions, requires fewer radiation tracks to induce cell death. The short path length of α-particles radiation confines its cytotoxic effect to the target tissue and the surrounding tumor microenvironment while limiting toxic effects to non-neoplastic tissues.

Following the successful clinical TAT with ^223^Ra dichloride for the treatment of metastatic castration-resistant prostate cancer with bone metastases and the clinical experience with ^213^Bi- and ^225^Ac-labeled compounds^2–4^, there has been an increased interest in new applications of TAT for many tumor types. Among the α-particles-emitting radionuclides for TAT, ^211^At can be produced using a cyclotron with an α-particle beam, and its separation and purification from the target has been established after decades of continuous research. After a branch decay with a half-life of 7.21 h, ^211^At completely emits α-particles with 100% probability. A few clinical applications of ^211^At for the treatment of malignant neoplasms have been reported^5,6^.

The failure of medical and radiological anti-cancer therapies is partially attributable to the heterogeneity of cancer. One of the mechanisms explaining cancer heterogeneity is the existence of cancer stem cells (CSCs)^7^. CSCs exhibit self-renewal activity and long-term proliferating capability^8^. The concept of neoplastic stem cells may explain the failure of various therapies to achieve long-lasting responses in patients^9^, because CSCs are suggested as a potential source of resistance to different types of anti-neoplastic drug. Anti-neoplastic drugs are considered to act on mature neoplastic cells rather than on CSCs in many neoplastic tissues. This is partially owing to the fact that these cells exhibit intrinsic resistance to both cytoreductive and cytostatic drugs^10^. Moreover, CSCs develop acquired drug resistance and thus differentiate into heterogeneous cells by producing more malignant subclones^11^. Circulating CSCs may be associated with an increased risk of metastases following initially effective therapy results with poor prognosis in many therapeutic trials^12^. All these issues facilitate the need for developing new treatment strategies that focus on the elimination of CSCs through which therapeutic efficiency and prognosis can be improved^9,13^. Therefore, CSCs have recently been recognized as a major target of anti-cancer therapy, and considerable effort has been made to identify novel CSC markers and target expression profiles in order to develop CSC-targeted therapy and response evaluation.

The C-X-C chemokine receptor type 4 (CXCR4) is known to be expressed in various CSCs, and is associated with tumorigenicity, angiogenesis, invasion, and chemoresistance^14^. CXCR4 transduces a signal by increasing intracellular calcium ion levels and enhancing mitogen-activated protein kinase 1(MAPK1)/MAPK3 activation. CXCR4 is detectable on neoplastic stem cells in solid tumors and in hematologic neoplasms, such as diffuse large B-cell lymphoma (DLBCL), multiple myeloma (MM), and acute myeloid leukemia (AML). Although CXCR4 is highly expressed on normal hematopoietic cells, its expression levels are particularly high in hematologic malignancies. Therefore, ^68^Ga-pentixafor has been clinically applied in CXCR4-directed positron emission tomography (PET) imaging in patients with lymphoproliferative diseases^15,16^. The CXCR4-directed PET imaging with ^68^Ga-pentixafor showed a low-level and heterogeneous receptor expression in solid cancers^17–19^.

CXCR4 regulates the motility and development of AML stem cells, and an increased expression level of CXCR4 is a prognostic marker for disease relapse or survival in AML^20,21^. Since a preserved bone marrow microenvironment including stem cells is considered the main cause for treatment failure and relapse, CXCR4-directed therapy would be potentially effective.

Radioimmunotherapy is suitable for hematological malignancies, because such malignancies are usually radiosensitive. Malignant lineage-specific cell surface antigens have been identified, and human anti-mouse antibodies are less likely formed in patients even in those undergoing treatment with murine antibodies. Furthermore, because α-particles have a high LET and a small path length, single-cell neoplasms such as AML are expected to be treated more efficiently with high-energy α-irradiation than with β-irradiation.

The aim of this study is to assess the feasibility of CSC-targeted radioimmunotherapy of AML using an ^211^At-labeled anti-CXCR4 monoclonal antibody (^211^At-CXCR4 mAb). We examined the pharmacokinetics and dosimetry in a human AML xenograft model in mice to assess the feasibility of the therapy concept. The pharmacokinetic analysis and dosimetry of ^125^I-CXCR4 mAb were carried out to estimate the radiation doses from ^211^At-CXCR4 mAb, which were then compared with the radiation doses obtained from the pharmacokinetics data of this mAb.

## Results

### **Immunohistochemical staining of human AML xenograft with CXCR4**

**Figure 1:**
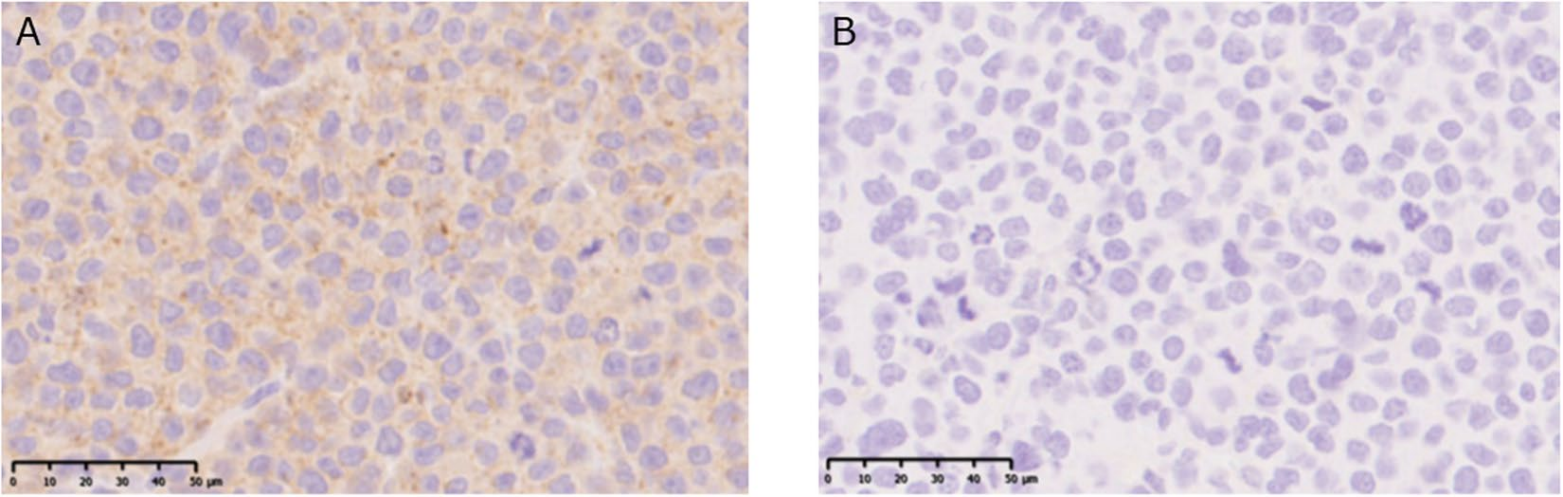
Immunohistochemical staining of CXCR4 in human acute myelogenous leukemia U937 xenografted mouse (**A**). Negative control (**B**).

The immunohistochemical staining of CXCR4 is shown in Fig. 1. Immunostaining showed both cytoplasmic and partially membranous staining of CXCR4 in the U937 tumor xenograft. The staining pattern was heterogeneous, and speckled high-intensity staining in the cytoplasm was observed.

### **Radiolabeling of anti-CXCR4 monoclonal antibody**

The radiochemical yields following the HPLC purification of [^125^I]SIB and [^211^At]SAB were 89.5% and 79.2% (decay-corrected), respectively. The yields of antibody conjugation with [^125^I]SIB and [^211^At]SAB were 30.0% and 18.6% (decay-corrected), respectively. The radiochemical purities and the specific activities just after purification of ^125^I-CXCR4 mAb and ^211^At-CXCR4 mAb were 96% and 93%, 0.116 MBq/μg and 0.189 MBq/μg, respectively.

### **Biodistribution study using**^**211**^**At-CXCR4 mAb and**^**125**^**I-CXCR4 mAb**

Table 1 summarizes the ^211^At-CXCR4 mAb and ^125^I-CXCR4 mAb uptakes in major organs and tumors up to 24 h after intravenous injection into the mice with tumor xenografts. ^211^At-CXCR4 mAb showed significantly lower %ID/g in blood than ^125^I-CXCR4 mAb from 1 to 24 h. From a rough calculation using blood samples measured at four time points, the biological half-lives of ^211^At-CXCR4 mAb and ^125^I-CXCR4 mAb in blood were 15.0 and 18.0 h, respectively. The blood clearance of ^211^At-CXCR4 mAb was comparatively faster than that of ^125^I-CXCR4 mAb. The same pattern of distribution was seen in the brain, kidney, and bone.

**Table 1:** Biodistributions of ^211^At-CXCR4 mAb and ^125^I-CXCR4 mAb in human AML xenograft model in athymic mice presented as percent injected radioactivity dose per gram of organ (%ID/g).

	^211^At-CXCR4 mAb				^125^I-CXCR4 mAb			
	1 min	1 h	6 h	24 h	1 min	1 h	6 h	24 h
Blood	37.87 ± 1.72	28.97 ± 1.98	20.38 ± 0.97	11.01 ± 1.26	41.32 ± 3.52	34.67 ± 3.19	24.28 ± 0.96	14.99 ± 1.78
Brain	0.89 ± 0.10	0.68 ± 0.04	0.47 ± 0.03	0.28 ± 0.04	0.96 ± 0.21	0.85 ± 0.06	0.60 ± 0.08	0.39 ± 0.04
Thyroid gland	6.06 ± 1.48	13.89 ± 3.52	21.46 ± 7.45	18.88 ± 6.33	2.13 ± 0.91	3.04 ± 0.71	4.23 ± 1.26	10.07 ± 0.80
Heart	4.77 ± 0.60	5.18 ± 0.43	5.57 ± 0.16	2.44 ± 0.27	4.37 ± 0.69	5.99 ± 0.61	6.24 ± 0.57	3.76 ± 0.17
Lung	14.85 ± 0.95	10.27 ± 0.19	8.19 ± 0.58	4.86 ± 0.35	10.32 ± 4.59	12.59 ± 1.37	9.13 ± 0.75	6.31 ± 0.25
Liver	7.82 ± 0.24	6.77 ± 0.31	4.39 ± 0.23	2.14 ± 0.32	6.69 ± 1.30	6.32 ± 0.98	5.06 ± 0.24	2.96 ± 0.10
Spleen	3.16 ± 0.31	7.01 ± 0.68	5.02 ± 0.44	2.65 ± 0.39	3.84 ± 1.16	6.35 ± 0.69	5.09 ± 0.33	3.03 ± 0.22
Pancreas	1.81 ± 0.14	1.40 ± 0.14	1.49 ± 0.19	1.23 ± 0.17	1.25 ± 0.28	1.15 ± 0.18	1.43 ± 0.13	1.66 ± 0.04
Stomach	1.46 ± 0.07	3.43 ± 0.11	4.68 ± 0.30	3.44 ± 0.43	1.14 ± 0.10	2.41 ± 0.36	2.92 ± 0.37	2.35 ± 0.17
Intestine	1.21 ± 0.08	2.50 ± 0.23	2.15 ± 0.26	1.37 ± 0.12	0.65 ± 0.10	2.45 ± 0.37	2.91 ± 0.20	1.55 ± 0.12
Kidney	7.37 ± 1.00	8.02 ± 0.64	5.17 ± 0.36	3.81 ± 0.46	7.30 ± 1.11	10.00 ± 1.07	8.04 ± 0.89	5.10 ± 0.28
Testis	0.57 ± 0.08	2.96 ± 0.13	4.33 ± 0.37	1.79 ± 0.14	0.50 ± 0.13	2.86 ± 0.19	4.15 ± 0.34	2.85 ± 0.13
Muscle	0.43 ± 0.04	0.53 ± 0.07	0.68 ± 0.18	0.97 ± 0.06	0.41 ± 0.06	0.55 ± 0.06	0.82 ± 0.09	1.26 ± 0.06
Bone	1.91 ± 0.09	2.54 ± 0.27	1.70 ± 0.07	1.39 ± 0.18	2.36 ± 0.39	3.35 ± 0.54	2.29 ± 0.15	1.75 ± 0.15
White fat	0.37 ± 0.11	0.69 ± 0.12	0.63 ± 0.09	0.88 ± 0.26	0.44 ± 0.18	0.65 ± 0.35	1.12 ± 0.25	1.26 ± 0.19
Tumor^#^	0.93 ± 0.06	3.91 ± 0.80	5.05 ± 2.49	3.04 ± 1.30	0.73 ± 0.15	2.99 ± 0.86	4.50 ± 0.33	4.43 ± 1.64
	(0.09 ± 0.11)	(0.09 ± 0.04)	(0.44 ± 0.28)	(0.57 ± 0.32)	(0.13 ± 0.12)	(0.09 ± 0.09)	(0.11 ± 0.13)	(0.14 ± 0.15)

^#^The weight of the tumor is shown in parentheses (mean ± SD).

Conversely, the thyroid gland and stomach showed significantly higher uptakes of ^211^At-CXCR4 mAb than ^125^I-CXCR4 mAb from 1 min to 24 h. The tumor showed gradually increased uptake of ^211^At-CXCR4 mAb, and the highest uptake was obtained at 6 h. ^125^I-CXCR4 mAb also showed the highest accumulation in the tumor at 6 h. Accumulation in other normal organs was almost similar for both ^211^At-CXCR4 mAb and ^125^I-CXCR4 mAb. The localizations of ^211^At-CXCR4 mAb and ^125^I-CXCR4 mAb in the tumor showed no significant difference.

**Figure 2:**
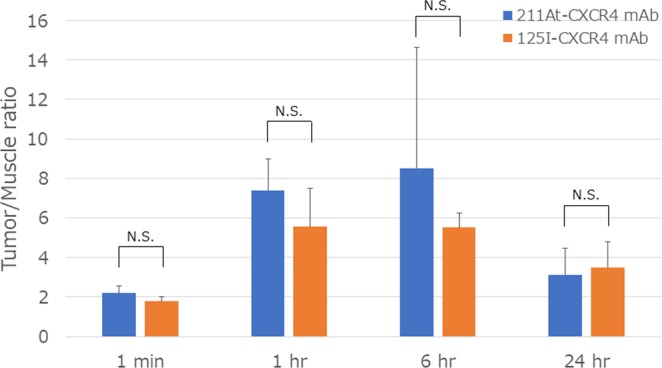
Tumor-to-muscle ratios of ^211^At-CXCR4 mAb and ^125^I-CXCR4 mAb uptake after intravenous administration in human AML xenograft model in athymic mice.

Compared with the background accumulation such as that in muscle, ^211^At-CXCR4 mAb was highly accumulated in the tumors (Fig. 2). The tumor-to-muscle ratio of ^211^At-CXCR4 mAb uptake was highest (8.51 ±6.14) at 6 h, at this time point, the tumor-to-muscle ratio of ^125^I-CXCR4 mAb uptake was 5.51 ± 0.74. The radioactivity in the thyroid gland was relatively high and gradually increased.

### Dosimetry of ^211^At-CXCR4 mAb in human AML xenograft model

Residence time estimation with the adult human phantom and an animal study with the murine xenograft model revealed that high accumulation of ^211^At-CXCR4 mAb activity was found in the lung, bone, bone marrow, and liver, with residence times of 0.330, 0.277, 0.277, and 0.272 Bq-h/Bq, respectively. The mean organ absorbed dose estimated with the 25 g mouse phantom was highest in the thyroid gland, followed by the stomach, lung, heart, and kidney for ^211^At-CXCR4 mAb. On the other hand, the absorbed dose estimated using the ^125^I-CXCR4 mAb was highest in the lung, followed by the kidney, heart, and stomach in decreasing order. The absorbed dose of ^125^I-CXCR4 mAb in the thyroid gland was lower than that of ^211^At-CXCR4 mAb.

The absorbed dose estimated using an adult male human phantom was highest (2.37 mGy/MBq) in the bone, followed by the thyroid, heart, bone marrow, testis, and lung in decreasing order (0.985, 0.773, 0.512, 0.492, and 0.399 mGy/MBq, respectively) (Table 2). The human absorbed doses of ^211^At-CXCR4 mAb estimated using the ^125^I-CXCR4 mAb are almost in agreement with those of ^211^At-CXCR4 mAb. However, the salivary gland, thyroid gland, and testis showed lower absorbed doses, whereas the adrenal gland and kidney showed higher absorbed doses of ^211^At-CXCR4 mAb estimated using ^125^I-CXCR4 mAb than those of ^211^At-CXCR4 mAb.

**Table 2:** Organ absorbed dose per unit administered activity (mGy/MBq) for ^211^At-CXCR4 mAb using adult male human phantom and biodistribution data of ^211^At-CXCR4 mAb and ^125^I-CXCR4 mAb in human AML xenograft model in athymic mice.

Organ	Absorbed dose (mGy/MBq)	
	^211^At-CXCR4 mAb	^125^I-CXCR4 mAb
Brain	0.024	0.032
Eye	0.087	0.098
Salivary gland	0.331	0.153
Thyroid gland	0.985	0.298
Heart	0.773	0.912
Lung	0.399	0.488
Thymus	0.088	0.099
Liver	0.220	0.248
Gallbladder	0.087	0.099
Spleen	0.244	0.247
Pancreas	0.073	0.079
Esophagus	0.088	0.099
Stomach	0.088	0.099
Small intestine	0.088	0.099
Right hemicolon	0.087	0.099
Left hemicolon	0.087	0.099
Rectum	0.087	0.098
Adrenal gland	0.193	0.291
Kidney	0.287	0.402
Urinary bladder	0.087	0.098
Testis	0.492	0.185
Prostate gland	0.087	0.098
Bone	2.370	2.980
Red marrow	0.512	0.627
Total body	0.118	0.136

**Figure 3:**
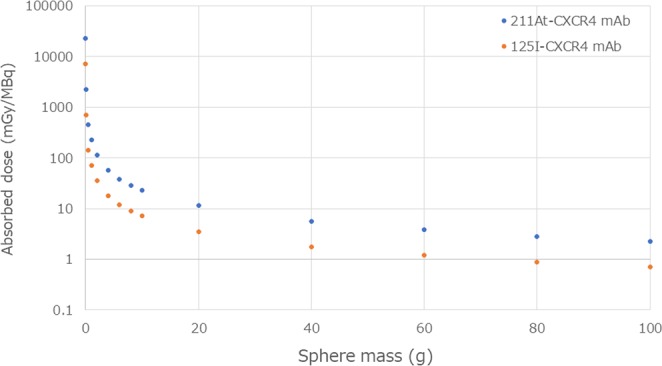
Mean absorbed dose calculations of ^211^At-CXCR4 mAb and ^125^I-CXCR4 mAb in tumor estimated using the unit-density sphere model.

The tumor absorbed dose estimated using the sphere model is shown in Fig. 3. The weights of the tumors obtained in the present biodistribution studies (0.299 ± 0.249 g for ^211^At-CXCR4 mAb and 0.116 ± 0.024 g for ^125^I-CXCR4 mAb) were used for the calculation. The threefold difference in the tumor absorbed doses between ^211^At-CXCR4 mAb and ^125^I-CXCR4 mAb may be explained by the difference in the weights of the tumors. The estimated absorbed doses of ^211^At-CXCR4 mAb were 22.8 mGy/MBq in the tumor of 10 g and 11.4 mGy/MBq in the tumor of 20 g. From the dosimetry analysis, the tumor-to-bone marrow ratio and tumor-to-kidney ratio of the absorbed doses were 44.5 and 79.4 for the tumor of 10 g, and 22.3 and 39.7 for the tumor of 20 g, respectively.

## Discussion

To examine the feasibility of α-particle therapy targeting CXCR4 in AML, a preclinical study was carried out with ^211^At-CXCR4 mAb and ^125^I-CXCR4 mAb in a human AML xenograft model. A biodistribution study revealed that ^211^At-CXCR4 mAb as well as ^125^I-CXCR4 mAb showed the highest tumor uptake and the highest tumor-to-muscle ratio at 6 h, which agreed with the physical half-life of ^211^At, although the precise biological behavior from 6 to 24 h was not evaluated in this study. Clinically effective α-particle-emitting isotopes for cancer therapy should have a reasonably short half-life, which will limit long-term radiation exposure and allow for the production, preparation, and administration of these isotope-labeled compounds for clinical applications. Although the effective half-life of whole IgG antibodies in the circulation has been considered to be longer than the physical half-life of ^211^At (7.2 h) as used in this study, in the case of AML, the reasonably slow clearance of ^211^At-CXCR4 mAb from blood may not be a disadvantage for the therapeutic effect on the stem cells as long as normal organ toxicity is tolerable, because AML stem cells may be present mainly in the bone marrow and partly in the circulation.

The estimated residence time indicated that ^211^At-CXCR4 mAb was present in the lung, bone, bone marrow, and liver at high concentrations for a certain duration, and mainly excreted by the hepatobiliary system as expected. A comparative study revealed that ^211^At-CXCR4 mAb showed a slightly faster clearance from blood and a higher uptake than ^125^I-CXCR4 mAb in the thyroid gland and stomach. This is at least partly explained by the instability of ^211^At binding to CXCR4 mAb. ^211^At was used for labeling anti-CXCR4 mAb by a standard two-step procedure; however, ^211^At has been found to be unstable after binding by this method^22^. *In vivo* deastatination has been reported to be attributable to the weaker carbon–halogen bond and oxidative dehalogenation for astatine than for iodine^23^.

Although the highest %ID/g in the tumor was acquired at 6 h after the administration of ^211^At-CXCR4 mAb, it was still lower than those in the lung, heart, and kidneys. This is explained by the results of immunohistochemical analysis as shown above and the data reported in the literature showing that a high level of staining is seen heterogeneously in the cytoplasm^20^. Moreover, the relatively low tumor uptake may be partly explained by the fact that CXCR4 is not a tumor-specific antigen.

The major hurdle of radioimmunotherapy is to deliver tumoricidal doses to tumors, while sparing the normal function of radiosensitive organs. Tumoricidal doses range from 30–50 Gy for radiosensitive tumors including hematopoietic neoplasms, and up to 100 Gy for radioresistant tumors. The tolerated radiation doses in normal organs such as the kidney, lung, colonic mucosa, and bone marrow are reported to be less than 20, 15, 2.5, and 1 Gy, respectively^24^. The present dosimetry analyses showed that the bone marrow was a potential dose-limiting organ with an absorbed dose of 0.512 mGy/MBq. Accordingly, the bone marrow absorbed dose of 0.512 mGy/MBq and the maximum tolerated dose of 1 Gy are assumed, the maximum administration dose is calculated to be 1.95 GBq. Then the tumor absorbed doses would be 44.5 and 22.3 Gy for tumors of 10 and 20 g, respectively. In this dose setting, the absorbed doses in the lung, kidney, and colon are 0.78, 0.56, and 0.17 Gy, respectively; these values are below the tolerated dose as mentioned above. However, the administration dose of 1.95 GBq calculated in this scenario is not realistic, because the biological effect of α-particles is not considered in the calculation of tolerated dose in normal organs. Although the relative biological effectiveness (RBE) of α-particles has not been determined, the following ways of considering the biological effect may be possible. From the ICRP Publication 92, the radiation weighting factor (w_R_ = 20) and tissue weighting factor (w_T_ = 0.12 for bone marrow) are expediently used for calculating the bone marrow tolerated dose as 1.23 mGy/MBq (0.512 × 20 × 0.12), and the maximum administration dose of 0.81 GBq and tumor absorbed dose of 18.5 Gy for a tumor of 10 g are obtained. Another calculation method is using an assumed RBE of 5; in this case, the maximum administration dose and tumor absorbed dose would be 0.39 GBq and 8.9 Gy, respectively.

It is essentially reasonable to estimate the absorbed dose of ^211^At-CXCR4 mAb using the biodistribution data of ^125^I-CXCR4 mAb, since a biodistribution study with ^211^At-labeled compounds is, in general, hardly performed in comparison with that with ^125^I-labeled compounds. Therefore, ^125^I-labeled compounds would be often used for the primary proof-of-concept study to assess the feasibility of a novel ^211^At-labeled compound. If image analysis is required, ^123^I-labeled compounds will be used. The biodistribution of a compound labeled with radioactive iodine, such as ^123^I and ^125^I, is assumed to be identical to that of an ^211^At-labeled compound. In this study, a biodistribution study was performed with ^125^I-CXCR4 mAb to estimate the dosimetry of ^211^At-CXCR4 mAb. The results revealed that major organs showed radiation doses almost similar to those estimated with ^211^At-CXCR4 mAb as a reference. However, doses in the thyroid gland, salivary gland, and testis were underestimated with ^125^I-CXCR4 mAb. The underestimation of the thyroid dose would be at least partly explained by the relative instability of ^211^At-CXCR4 compared with that of ^125^I-CXCR4 mAb.

The selective targeting of tumors relative to normal tissues is the key principle of targeted radionuclide therapies including TAT. Therapeutic index (TI) or the ratio of radiation absorbed dose in the tumor to the absorbed dose in radiosensitive tissues, such as the bone marrow and kidney, is important for evaluating the feasibility of a targeted radionuclide therapy. Pharmacokinetic evaluation and dosimetry analyses of ^211^At-CXCR4 mAb revealed that the TIs, tumor-to-bone marrow and tumor-to-kidney, for the tumor of 10 g, were 44.5 and 79.4, and the TIs for the tumor of 20 g were 22.3 and 39.7, respectively. The preferable TIs, tumor-to-bone marrow and tumor-to-kidney are >50 and >10, respectively; however, AML does not usually form tumors, and AML cells as well as AML stem cells are present as single cells in the circulation. Although the sphere model used in this study could not be applied to the dosimetry of a single cell, the target cell-to-bone marrow ratio must be much greater than 44.5. Therefore, the present estimation shows a possible therapeutic efficacy for AML.

The limitation of α-particle dosimetry carried out in this study should be mentioned. Owing to the short path length of α-particles, the heterogeneous distribution of radiopharmaceuticals in the tissue and the target-cell-related geometry are important for estimating cellular damage caused by DNA breaks. Therefore, it is inaccurate to calculate the absorbed dose of α-particles, instead of β-particles, by the MIRD formalism. For the accurate dosimetry calculation of α-particles, data of radioactivity distribution as a function of time at the cellular and subcellular levels are required. The nonuniform distribution of radionuclides attributable to the heterogeneous expression of target molecules as indicated in the results of immunohistochemistry leads to a variable spatial distribution of conjugates and a complicated source–target configuration. Spatial and temporal changes in the distribution of ^211^At-CXCR4 mAb in the tumor should be accurately measured for accurate dosimetry. High-resolution detectors, such as α-cameras and microscale pharmacokinetic models in the tumor microenvironment, should be developed^25^. However, there is no established microscale dosimetry method to estimate the absorbed dose of α-particles at the cellular level; thus, conventional dosimetry analysis was carried out to estimate the α-particle absorbed dose in this study. Therefore, the absorbed doses in the tumor and organs were only estimated values in this study. The lack of a non-specific control used in the animal study and evaluation of CXCR4 expression on stem cells is another limitation.

CXCR4-directed therapies using either alternative small-molecule CXCR4 antagonists^26,27^, CXCL12 inhibitors^28^, or anti-CXCR4 antibodies^29,30^ have resulted in prolonged overall survival in preclinical studies. The use of gold nanoparticles labeled with ^188^Re targeting CXCR4 has been studied to reverse chemoresistance of CSC in glioblastoma^31^.

Recently, clinical experience with ^177^Lu- and ^90^Y-labeled pentixather for the CXCR4-targeted radionuclide therapy for advanced-stage DLBCL, MM, and AML, has been reported^32,33^. The latest report at present showed a partial response in two of six patients who underwent additional radioimmunotherapy with a ^188^Re-anti-CD66 antibody or ^90^Y-anti-CD20 antibody (ibritumomab tiuxetan)^33^. In these studies, radionuclide therapies were performed in addition to high-dose chemotherapy, followed by allogeneic hematopoietic stem cell transplantation. Radionuclide therapy without hematopoietic stem cell rescue might be preferable for patients to preserve their own hematopoietic progenitor cells in the bone marrow, as long as radionuclide therapy is investigated for curative intent.

In summary, analyses of the pharmacokinetics and dosimetry of ^211^At-CXCR4 mAb in the tumor xenograft revealed that the clearance from blood and the tumor uptake matched the physical half-life of ^211^At, and the target cell-to-normal organ ratios of absorbed dose would be high enough to eradicate tumor cells, although tumor uptake was relatively low. This study suggests the possibility of stem-cell-targeted α-particle therapy using ^211^At-CXCR4 mAb for AML and supports further evaluation by therapeutic studies.

## Methods

### **Cell line and xenograft model**

The human AML cell line U937 was purchased from the European Collection of Authenticated Cell Cultures (Salisbury, UK). U937 cells were cultured at 37 °C in a humidified atmosphere of 5% CO_2_ in RPMI 1640 medium (Invitrogen/Thermo Fisher Scientific, Inc., Carlsbad, CA, USA), supplemented with 10% heat-inactivated fetal bovine serum (Nichirei Bioscience, Tokyo, Japan), 100 units/ml penicillin, and 100 μg/ml streptomycin (Sigma-Aldrich, St. Louis, MO, USA).

The entire experimental protocols were approved by the Laboratory Animal Care and Use Committee of Fukushima Medical University (approval number 28071) and performed in accordance with the Guidelines for Animal Experiments at Fukushima Medical University. Eight-week-old male athymic BALB/c nude mice were purchased from Japan SLC, Inc. (Hamamatsu, Japan) and maintained in a specific-pathogen-free animal experiment facility. The room temperature was maintained between 23 and 25 °C, and the relative humidity was maintained between 45 and 55%. The institutional laboratory housing provided a 12-h light/dark cycle and met all criteria of the Association for Assessment and Accreditation of Laboratory Animal Care (AAALAC) International (http://www.aaalac.org/). To prepare a tumor xenograft, a suspension of 1 × 10^7^ cells in 100 μL PBS(–) was injected subcutaneously in the dorsal flank of the mice, and the mice were grown for 2–3 weeks (size approximately 200–300 mm^3^).

### **Production and purification of**^**211**^**At**

**Figure 4:**
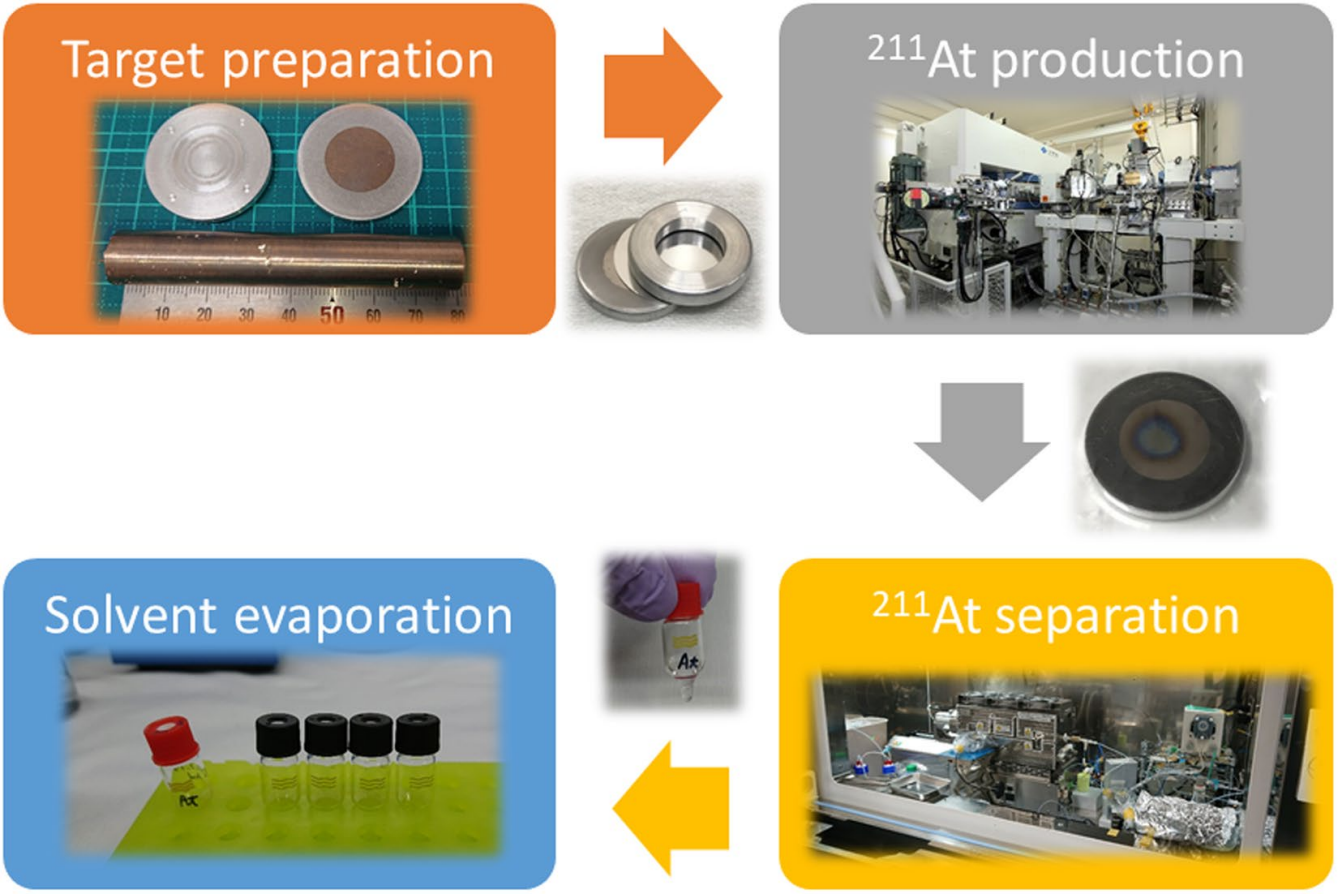
Schematic diagram of production, separation, and preparation of ^211^At for radiolabeling of anti-CXCR4 mAb.

^211^At was produced by ^209^Bi(α, 2n)^211^At nuclear reaction using a MP-30 cyclotron (Sumitomo Heavy Industries Ltd. Tokyo, Japan) in our facility^34^. A schematic diagram of production, separation, and preparation of ^211^At for the radiolabeling of anti-CXCR4 mAb is shown in Fig. 4. The bismuth (Bi) target was purchased from Nilako (Tokyo, Japan) and its purity was 99.9999%. The target was placed on the irradiation system connected to the end of the beamline. An α-particle beam of 30 MeV was degraded to 28.6 MeV by inserting aluminum foil of 30 μm attached in front of the target to prevent the production of ^210^At. The degraded beam was bombarded to a 0.1-mm-thick and 18-mm-diameter Bi layer on a 1-mm-thick and 32-mm-diameter aluminum backing plate for 1.5 h with 20 eμA. During the irradiation, surface and bottom of the target were cooled by helium gas flow and water circulation, respectively. The radioactivity of ^211^At was 820 MBq at the end of the bombardment.

^211^At was isolated from the irradiated target materials by the dry distillation method proposed by Lindegren *et al*. with slight modifications^35^. Briefly, the target was inserted in a quartz tube placed in a tube furnace preheated to 800 °C. Under helium gas flow in an 850 °C oven, vaporized ^211^At was transported from the quartz tube to an externally connected PTFE tube which was immersed in a dry ice/ ethanol (EtOH) bath. The cooled ^211^At was trapped in a PTFE tube then eluted and recovered using 0.5 mL of chloroform. The radioactivity after purification was 390 MBq. The resulting solution was placed in a 1.1-mL-volume glass vial, and the solvent was removed using heat and a gentle stream of N_2_ gas. Finally, ^211^At was prepared in dry form in a glass vial.

The activity of ^211^At was measured using a dose calibrator (CRC-25R, Capintec Inc., Ramsey, NJ, USA) before and after the dry distillation. Gamma-ray spectrometry was also performed using a Ge detector (GEM30–70, ORTEC, Oak Ridge, TN, USA) to assign the radionuclide produced in the target and the final solution of ^211^At. The radionuclidic purity of ^211^At was more than 99.9% at the end of recovery.

### **Radiolabeling of anti-CXCR4 monoclonal antibody**

The BSA- and azide-free anti-human rabbit monoclonal antibody against CXCR4 (clone UMB2, ab197203, Abcam, Cambridge, UK) was used for radiolabeling in this study. [^125^I]Sodium iodide (644 GBq/mg as iodine) was purchased from PerkinElmer (Waltham, MA, USA). *N*-Succinimidyl 3-trimethylstannyl-benzoate (*m*-MeATE) was purchased from Toronto Research Chemicals (North York, Canada). Other reagents were of reagent grade. All chemicals obtained commercially were used without further purification. HPLC analyses were performed on Shimadzu Prominence series coupled to an SPD-M20A photodiode array UV detector (Shimadzu Corporation, Kyoto, Japan) or a Gabi gamma detector (Elysia-Raytest, Straubenhardt, Germany). LabSolutions Software (Shimadzu Corporation, Kyoto, Japan) was used for controlling the HPLC system and processing data.

CXCR4 mAb was labeled with ^125^I and ^211^At as follows. *N*-Succinimidyl 3-[^125^I]iodobenzoate ([^125^I]SIB) and *N*-succinimidyl 3-[^211^At]astatobenzoate ([^211^At]SAB) were prepared by a method reported previously with slight modifications^36,37^. [^125^I]SIB and [^211^At]SAB were purified by RP-HPLC on Cosmosil 5C_18_-AR-II (4.6 × 150 mm, Nacalai Tesque, Kyoto, Japan) at a flow rate of 1 mL/min with the gradient mobile phase of 40% acetonitrile in water with 0.1% TFA to 60% acetonitrile in water with 0.1% TFA for 20 min. Fractions containing [^125^I]SIB or [^211^At]SAB were collected, and the solvent was evaporated.

[^125^I]SIB was added to 50 µL of anti-CXCR4 mAb (40 µg) in 0.25 M borate buffer (pH 8.5) and reacted on ice for 2 h. [^211^At]SAB was added to 50 µL of anti-CXCR4 mAb (50 µg) in 0.25 M borate buffer (pH 8.5) and reacted on ice for 1 h. ^125^I-CXCR4 mAb and ^211^At-CXCR4 mAb were purified using a PD-10 column (GE Healthcare, Uppsala, Sweden) with saline. The radiochemical purity of ^125^I-CXCR4 mAb was measured by instant thin-layer chromatography (ITLC) using a Tec-control^TM^ 150–771 strip (BIODEX, Shirley, NY, USA). The strip was eluted with a 70% acetone/30% water mixture^38^. The radiochemical purity of ^211^At-CXCR4 mAb was measured by methanol precipitation because free astatine could not be separated from ^211^At-CXCR4 mAb using ITLC^39^. Radioactivity was measured with an automated well gamma counter (2480 WIZARD^2^, PerkinElmer Inc., Waltham, MA, USA).

### **Immunohistochemical staining of human AML xenograft with CXCR4**

The formalin-fixed, paraffin-embedded U937 tumor tissues were sectioned at 3 μm thickness. Immunohistochemical staining of CXCR4 was performed using paraffin-embedded sections by a polymer peroxidase method (Envision + /horseradish peroxidase; Dako Cytomation A/S, Glostrup, Denmark). Briefly, deparaffinized, rehydrated sections of U937 tumor resected from xenografts were immersed in a water bath at 95 °C for 30 min in TE buffer solution (1 mmol/L EDTA including 10 mmol/L Tris-buffered saline of pH 9.0) and cooled for 20 min to room temperature. After rinsing with Tris-buffered saline (0.05% Tween/0.15 mol/L NaCl including 10 mmol/L Tris-buffered saline of pH 7.6), the sections were treated with 0.3% hydrogen peroxide in Tris-buffered saline to block endogenous peroxidase activity, and then incubated in normal horse serum for 3 min to block nonspecific antibody binding. Then, the sections were incubated for 60 min at room temperature with affinity-purified anti-human rabbit monoclonal antibody against CXCR4 (clone UMB2, ab124824, Abcam, Cambridge, UK) at a dilution of 1:200. Thereafter, they were incubated with Histofine^®^ Simple Stain MAX-PO (MULTI) (Nichirei Biosciences Inc., Tokyo, Japan) and 0.02% 3,3′-diaminobenzidine tetrahydrochloride as the chromogen. Finally, nuclear counterstaining was carried out with hematoxylin (Sakura Fine Tek Japan Co., Ltd., Tokyo, Japan). For negative control, the incubation step with the primary antibody was omitted.

### **Biodistribution study with**^**211**^**At-CXCR4 mAb and**^**125**^**I-CXCR4 mAb**

In the animal experiments, the nonradiolabeled antibody and saline were added to adjust the amount to 5 μg of antibody/100 μL. ^211^At-CXCR4 mAb of 320 kBq/5 µg antibody in 100 μL PBS(−) or ^125^I-CXCR4 mAb of 60 kBq/5 µg antibody in 100 μL PBS(−) were injected intravenously into the mice. When the tumors were 8–15 mm in long axis, groups of 4 and 5 mice were used for the ^211^At-CXCR4 mAb and ^125^I-CXCR4 mAb experiments, respectively. At 1 min, 1 h, 6 h, and 24 h after injection, the mice were euthanatized, and aliquots of blood were collected. The organs and tumor were excised from each mouse and weighed, and radioactivity was measured with an automated well gamma counter. The radioactivities of the organs, blood, and tumor are presented here as percent injected radioactivity dose per gram (%ID/g). Because the weight of the thyroid gland could not be correctly measured, the standard weight of 0.014 g was applied.

### **Dosimetry of**^**211**^**At-CXCR4 mAb in human AML xenograft model**

Dosimetric analysis was performed with pharmacokinetic data from the murine biodistribution studies of ^211^At-CXCR4 mAb and ^125^I-CXCR4 mAb incorporated into the OLINDA/EXM ver. 2.0 code (Hermes Medical Solutions, Stockholm, Sweden)^40^. Biodistribution data of ^125^I-CXCR4 mAb were used for the estimation of ^211^At-CXCR4 mAb dosimetry. The mean doses per unit of injected activity (mGy/MBq) of α-decay from ^211^At absorbed by each organ and tumor were estimated in accordance with the standard method using the MIRD formalism and S-values^40–42^.

The mean radioactivities in organs at 1 min, 1 h, 6 h, and 24 h for each group of mice were used to calculate residence times (Bq-h/Bq) for extrapolating into every human organ based on the difference in tissue-to-body weight ratio between the mice model and the human phantom^43^. The total body weight and organ weight of mice were measured. The human body weight of 73.0 kg and organ weight were obtained from the reference adult male phantom in the OLINDA/EXM ver. 2.0.

The accumulated activity in the bone marrow was calculated from the accumulated activity in the blood using the red marrow-to-blood ratio (RMBLR), which has been validated using the bone marrow aspiration data of radiolabeled antibodies, and the RMBLR of 0.36 was used in this study^44^.

The tumor absorbed dose was calculated using the OLINDA/EXM ver. 2.0 unit-density sphere model. Time-integrated activity was obtained on the basis of the tumor uptakes of ^211^At-CXCR4 mAb and ^125^I-CXCR4 mAb in the biodistribution study as mentioned above to integrate the activity in the tumor over time.

### Statistical analyses

Continuous measures are presented as mean ± standard deviation. Average values were compared using the independent sample t-test and Mann–Whitney U-test. P-values <0.05 were considered statistically significant. Statistical calculation was carried out using GraphPad Prism 8 (GraphPad Software, San Diego, USA).

## Data Availability

The data generated and/or analyzed during the current study are available from the corresponding author on reasonable request.
